# PhenoMetaboDiff: R Package for Analysis and Visualization of Phenotype Microarray Data

**DOI:** 10.3390/genes15111362

**Published:** 2024-10-24

**Authors:** Rini Pauly, Mehtab Iqbal, Narae Lee, Bridgette Allen Moffitt, Sara Moir Sarasua, Luyi Li, Nina Christine Hubig, Luigi Boccuto

**Affiliations:** 1 JC Self Research Institute, 106 Gregor Mendel Circle, Greenwood, SC 29646, USAlboccut@clemson.edu (L.B.); 2 Greenwood Genetic Center, 106 Gregor Mendel Circle, Greenwood, SC 29646, USA; 3 School of Computing, Clemson University, 821 McMillan Rd, Clemson, SC 29634, USAnhubig@clemson.edu (N.C.H.); 4 School of Nursinghl, Clemson University, 414 Edwards, Clemson, SC 29634, USA

**Keywords:** microarrays, metabolic profiles, computational analysis

## Abstract

Background: PhenoMetaboDiff is a novel R package for computational analysis and visualization of data generated by Biolog Phenotype Mammalian Microarrays (PM-Ms). These arrays measure the energy production of mammalian cells in different metabolic environments, assess the metabolic activity of cells exposed to various drugs or energy sources, and compare the metabolic profiles of cells from individuals affected by specific disorders versus healthy controls. Methods: PhenoMetaboDiff has several modules that facilitate statistical analysis by sample comparisons using non-parametric Mann–Whitney U-test, the integration of the OPM package (an R package for analysing OmniLog^®^ phenotype microarray data) for robust file conversion, and calculation of slope and area under the curve (AUC). In addition, the built-in visualization allows specific wells to be visualized in selected pathways for a particular time slice. Results: Compared to the standard OPM package, the features developed in PhenoMetaboDiff assess metabolic profiles by employing statistical tests and visualize the dynamic nature of the energy production in several conditions. Examples of how this package can be used are demonstrated for several rare disease conditions. The incorporation of a graphical user interface expands the utility of this program to both expert and novice users of R. Conclusions: PhenoMetaboDiff makes the deployment of the cutting-edge Biolog system available to any researcher.

## 1. Introduction

The recent advancement in next-generation sequencing technologies has led to a dramatic increase in the detection of genetic variants and consequently in the diagnostic yield for disorders where the genetic etiology is not well characterized. However, the augmented detection of novel variants calls for functional validation of candidate genes. High-throughput transcriptomic and proteomic assays have allowed for a broad investigation of the functional impact of variants, but a more refined approach is necessary to access the metabolic effects of genetic variants. Two general types of metabolomic assessments are used: measurements of circulating metabolites and cell-based approaches. Comprehensive strategies have been developed to analyze the data generated by metabolomic assays based on the assessment of circulating levels of various metabolites [1]. However, a similar strategy would be beneficial for cell-based metabolic studies because metabolic profiling has allowed the characterization and identification of markers for altered metabolic pathways and biomarker discovery [2,3,4]. Analytic approaches are not as well developed for cell-based metabolic profiling. These metabolic profiling studies utilized the Biolog Phenotype Mammalian Microarray (PM-M) technology to generate data based on cells’ energy production in different metabolic environments. The system uses plates with 96 wells, each containing a different substrate; cell energy production is measured colorimetrically and data for each well are generated. Such data can be analyzed by the OPM package [5] offered by Biolog and open-source tools, but the analysis is cumbersome and requires advanced programming skills. The complexity has limited the usability of the OPM package. Still, such approaches present intrinsic limitations, such as the impossibility of comparing data from wells within the same array or the lack of customized reports and visuals that can address specific goals of individual projects. Metabolic profiling of different cell types represents a promising in vitro model to investigate the functional effects of multiple variants on multiple metabolic pathways. For metabolic profiling to be most useful, analysis methods need to be readily available and usable for researchers without relying upon highly trained programmers to develop custom analysis pipelines. Developing a more user-friendly analytic tool that could address the limitations of the available software packages will dramatically benefit the investigators and, ultimately, the patients.

To address the shortcomings mentioned above, we developed PhenoMetabodiff, a novel R package for computational analysis and data visualization generated by Biolog PM-Ms. This tool includes a simple data import utility within each module.

The data import utility uses a standard browse feature to allow the user to find and upload the standard data file produced by the Biolog system. The import utility handles both CSV and XLSX file types and allows the user to preview the data. The tab for each of the three modules includes its own data import utility.

- PhenoDiff Module: The main functionality of this module is to identify significant differences in the utilization of metabolites between two comparison groups using the non-parametric Mann–Whitney U-test.
- Kinetic Analysis: The Kinetic Analysis module plots a kinetic profile of the substrate utilization over time. These kinetic values can then be used to construct metabolic profiles of individual substrates over time. The user-defined feature can be used to visualize absorbance differences/overlap between two groups by either selecting specific wells or by customizing the interval time.
- Slope and AUC Calculator: The “Slope and AUC” module calculates the descriptive curve parameters, including lag phase, steepness of the slope, maximum curve height, and area under the curve, AUC.

The purpose of this manuscript is to describe the functionality of the new software PhenoMetaboDiff (v1.0.0) and to demonstrate its utility with examples from three recent applications, even though the software has been successfully employed in numerous other studies focused on metabolic profiling of genetic and complex disorders [6,7,8,9,10,11]. In the remainder of this paper, we discuss the materials and methods used and the software engineering that took place to generate this package.

## 2. Materials and Methods

### 2.1. Data

For demonstration purposes, modules in PhenoMetaboDiff were applied to the analysis of three separate sets of patient samples:

1. PhenoDiff on Autism Spectrum Disorder (ASD) [2].
2. Kinetic Analysis on Kosaki Overgrowth Syndrome [4]
3. Slope and AUC Calculator on Snyder–Robinson Syndrome (SRS) [3].

Informed consent was approved by the Self Regional Healthcare Institutional Review Board (IRB) for Human Research and was reviewed and signed by all the participants evaluated and their legal guardians. The IRB approved the use of the samples in the studies reported in this paper. Clinical and genetic features of the patients enrolled in the three studies were discussed in [2,3,4], as well as the data generated via the OPM package (v1.3.77).

### 2.2. Description of the Biolog Metabolic Arrays and Customized Plates

A description of the cutting-edge Biolog system is needed to understand the importance of the back-end analysis performed by PhenoMetaboDiff. The Phenotype Mammalian MicroArray (PM-M), developed by Biolog Inc.(Hayward, CA, USA), is designed to provide an unbiased scan of the activity of cellular metabolic pathways involved in the rate of production of NADH. Each well contains a single chemical as the sole energy source, and production of NADH per well is monitored. The Omnilog^®^ system collects optical density reads every 15 min, generating 96 data points for each well. The system also generates a kinetic curve for the metabolic reaction in each well and extrapolates parameters such as the slope, highest point, endpoint, the area under the curve (AUC), and lag. From previous observations, AUC resulted in the most comprehensive and informative parameter to assess the kinetics of the metabolic reaction of the cell lines. At the end of the final 24 h incubation with the Biolog Redox Dye Mix MB, the plates were analyzed utilizing a microplate reader with readings at 590 and 750 nm. The first value ($A_{590}$) indicated the highest absorbance peak of the redox dye, and the second value ($A_{750}$) gave a measure of the background noise. The relative absorbance ($A_{590–750}$) was calculated per well with values ranging between 0 (minimum) and 2 (maximum.)

### 2.3. Components of PhenoMetaboDiff

#### 2.3.1. Implementation

The infrastructure for the tool was created using the open-source programming language R (v4.1.2) (http://www.r-project.org, accessed on 2 August 2024) for the analytical modules, Perl (v5.40.0) (http://www.perl.org, accessed on 2 August 2024) for parsing the Omnilog^®^ PM-M data, and finally, Shiny R (v1.9.1) was used for the graphical user interface (GUI). The code is available online: https://anonymous.4open.science/r/phenoMetaboDiff-62D4 (accessed on 2 August 2024).

#### 2.3.2. Data Processing and Statistical Analysis

**Figure 1:**
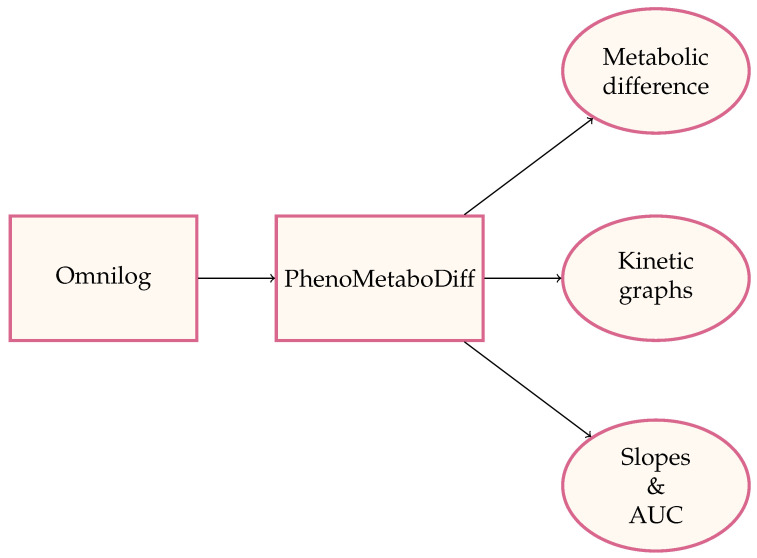
Workflow diagram for the analysis process using PhenoMetaboDiff.

For the PM-M data, the endpoint absorbance values were read into a CSV file. This CSV file is read into our first module, PhenoDiff. The goal of this module is to identify compounds that are differentially metabolized by patient versus control cells, as measured by NADH levels in each well of the 96-well plate. In our example, we compared people with autism spectrum disorder with age-matched controls. Non-parametric U-tests are run to obtain Benjamini–Hochberg (BH) adjusted p-values once the user-defined options, such as logarithmic transformation, are selected. The Mann–Whitney U-test was used because it is a robust non-parametric method to compare distributions without requiring that the samples be normally distributed, as is the assumption for the t-test. When sample sizes are small, the Mann–Whitney U-test is more conservative than the t-test. The kinetic table stores the time-point data for each of the phenotypes in the OmniLog^®^ screen. Data from the OmniLog^®^ instrument are exported as comma-separated values (CSVs) to upload into the system. Upon loading, the user links the kinetic dataset to each entry and a set of phenotypes. For search and analytical functionality, the initial landing screen of the application presents a choice of optional views. The user can select the search criteria of interest and then drill down following a selection hierarchy. In cases where replicates have been generated, the user can choose to average all phenotype replicates for the same experiment. The averaged result file can serve as an input in the PhenoDiff tab. Each Tab describes the input and output file structure. Once the records have been generated, the analysis modules are demonstrated to the user for further selections (Figure 1). The following outline describes each of the modules:

#### 2.3.3. PhenoDiff

**Figure 2:**
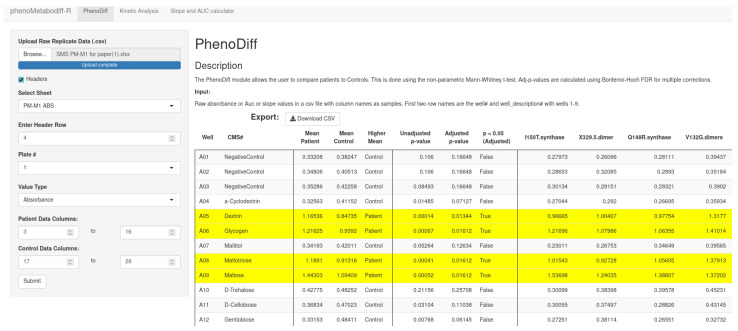
PhenoDiff allows the comparison of two groups using the non-parametric Mann–Whitney U-test. In this case, we compared patients with ASD to controls by uploading CSV files. The user specifies which columns belong to patients or controls. Additionally, the user can select several analysis features, such as the type of place, and types of analysis values, such as endpoint absorbance, slope, and AUC. In this case, rows with adjusted *p* value less than 0.05 are highlighted.

PhenoDiff (Figure 2) is an analytical functionality for conducting a non-parametric Mann–Whitney U-test between two groups of samples. This module includes the CSV data file, selecting the plate# to identify the correct normalization factor. In addition to a CSV data file, PhenoMetaboDiff is also able to read Microsoft’s XLSX files. When reading a Microsoft Excel file, the software allows the user to choose the appropriate sheet. As a default setting, the first sheet is selected. After loading the CSV or XLSX file, a preview is shown on the selected sheet so the user can check the validity of the input data. Following that, the user is also able to select the header to offset dynamically.

#### 2.3.4. Kinetic Analysis

**Figure 3:**
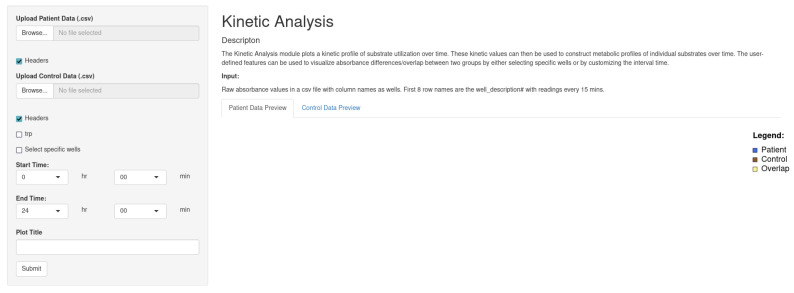
The kinetic analysis tab allows the user to designate a start and end time to generate a series of pathways taken by the well.

The Kinetic Analysis module (Figure 3) plots a kinetic profile of substrate utilization over time. These kinetic values can then be used to construct metabolic profiles of individual substrates over time. The user-defined features can be used to visualize absorbance differences/overlaps between two groups by either selecting specific wells of interest or by customizing the time intervals.

#### 2.3.5. Slope and AUC Calculator

The Slope and AUC Calculator module calculates descriptive curve parameters, lag phase, steepness of the slope, maximum curve height, and area under the curve, AUC, using the R package OPM. To compute the aforementioned results, the module takes raw absorbance values as input. The input is a CSV file with column names as corresponding wells. The first eight rows are the well description numbers with readings every 15 min.

The module also acts as an auxiliary to the Kinetic Analysis module by behaving as an exporting tool for the results of the kinetic analysis. The slope and AUC in the output CSV file can be the slope and AUC of the kinetic graph, given the same input data are used in both modules.

**Figure 4:**
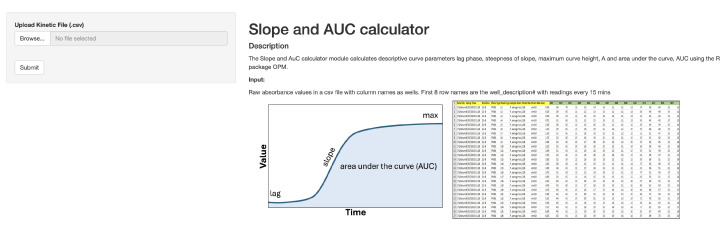
The Slope and AUC Calculator module calculates descriptive curve parameters, such as the lag phase, steepness of the slope, maximum curve height, and area under the curve, AUC, using the R package OPM (v1.3.77) [5].

The initial interface of the module is shown in Figure 4. The output of the module is another CSV file with all the values computed and formatted neatly to be imported into external tools for further analyses. This module was used along with the Kinetic Analysis module for the data from the patient with Kosaki overgrowth syndrome.

### 2.4. Demonstration Example: Kinetic Analysis of NADH

In order to demonstrate the utility of our package, we use two cases. For the first use case, we utilize data from a rare disorder, Kosaki overgrowth syndrome, characterized by distinctive facial features, brain white matter lesions, and developmental delay, as described in a previous paper [4]. Here, we consider data from a 19-year-old patient with Kosaki overgrowth syndrome. The Phenotype Mammalian MicroArray (PM-M) system was used to scan the activity of the cellular metabolic pathways involved in the rate of production of NADH (nicotinamide adenine dinucleotide, reduced form) as previously described [12]. For this demonstration, we will utilize the PM-M7 plate, which contains diverse metabolic effectors, including platelet-derived growth factor (PDGF-AB), as well as other growth factors targeting the same PI3K-AKT-mTOR pathway (insulin, IGF-1, FGF-1) or other pathways regulating cell growth and proliferation (human growth hormone, hGH). Each well contains a single compound as the sole metabolic effector, and production of NADH per well is monitored using colorimetric redox dye chemistry. The PM-M7 plate was incubated with 20,000 viable cells per well in a volume of 50μL. The readings were recorded every 15 min for 24 h. Kinetic curves of the NADH generation for each well were generated, and the relative absorbance ($A_{590–750}$) was calculated per well. By utilizing the kinetic data from the Biolog plate, we were able to visualize the metabolic differences.

Kinetic readings of the optical density representing the quantity of NADH generated for those wells containing increasing concentrations of PDGF revealed decreased production of NADH in the patient as compared to controls in a dose-dependent manner. Thus, the kinetic analysis feature clearly provided functional evidence for the pathogenic role of the variant under study.

**Figure 5:**
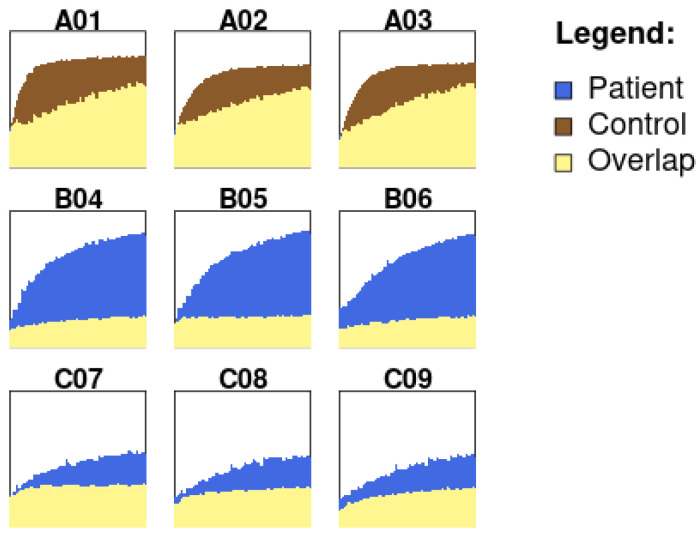
Visualization showing the results of the kinetic analysis of the NADH and the relative absorbance ($A_{590–750}$).

In the second case study, we establish the utilization of PhenoMetaboDiff to visualize spermine synthase deficiency caused by lysosomal dysfunction and oxidative stress in models of Snyder–Robinson syndrome—a slice of the resulting set of visualizations is shown in Figure 5.

## 3. Results

### 3.1. Resources and Innovation

The PhenoMetaboDiff package has been designed to facilitate the analysis and interpretation of high-throughput metabolomic data utilizing the Biolog PM-M technology. As compared to previous packages, like the OPM (v1.3.77) software used by default for PM-M data, this package is quicker, more straightforward in its passages, allows for the comparison of multiple plates and at the same time offers the option of analyzing and comparing individual wells instead of the entire 96-well plate. As highlighted in the examples reported in the Materials and Methods section, values from individual wells may be critical to identifying metabolic biomarkers, and it may be possible to compare the NADH production of two or more wells from the same plate. For example, D,L-lactic acid is in the G2 well of PM-M1 and lactic acid is in the G5 well of the same array; utilization of these two compounds reflects the efficacy of the anaerobic and aerobic metabolism, respectively. A comparison of the values in these two wells will provide fundamental information on the metabolic status of the cells, the mitochondrial activity, and eventually, a pathological shift from an aerobic prioritization of the energy-producing pathways to an anaerobic one, as observed in the Warburg effect, a phenomenon described in several types of cancer cells, which preferentially utilize anaerobic glycolysis instead of mitochondrial oxidative phosphorylation to generate the energy needed for cellular processes [13].

### 3.2. Translational Impact

The features offered by PhenoMetaboDiff allow a rapid and precise interpretation of metabolic data for various types of conditions, from complex multifactorial disorders like ASD [14,15,16,17,18,19] to monogenic diseases like Kosaki overgrowth [20,21,22] and Snyder–Robinson syndrome [23,24,25,26]. Such straightforward interpretation is critical for the employment of PM-M data in both research and clinical protocols because it provides a fundamental step for a standardized protocol that can be easily replicated in different laboratories. Metabolic profiles generated by PM-M data can lead to the identification of pathogenic mechanisms, as in the case of the subject with Kosaki overgrowth syndrome; an unambiguous detection of abnormal metabolic traits by Kinetic Analysis allowed us to propose disruption of specific metabolic pathways as a candidate underlying mechanism responsible for several clinical traits of the syndrome [4]. The analysis generated by PhenoDiff on ASD data has led to the identification of potential biomarkers for a relatively common disorder [2]. The main characteristics of an ideal biomarker are accuracy, reliability, sensitivity, specificity, rapid turnaround time, and low cost. A user-friendly software such as PhenoMetaboDiff contributes to many of these features: it validates the accuracy of the PM technology, warrants the reliability and replicability by using standardized methods for the analysis, allows for easy monitoring of the sensitivity and specificity of the data, and reduces time and costs because it does not require specialized professionals to analyze the PM data. The thorough analysis of multiple parameters guaranteed by PhenoMetaboDiff with the Slope and AUC Calculator allows researchers to explore different characteristics of metabolic processes, as observed in our study of cells from individuals with SRS [3]. Our analysis highlighted novel abnormalities in a rare disorder such as SRS, suggesting new potential targets for the development of therapeutical approaches. Overall, PhenoMetaboDiff represents an innovative tool that facilitates the application of metabolomic analyses to research projects and clinical practice by simplifying data analysis and interpretation.

### 3.3. Functional Studies

PhenoMetaboDiff has been utilized in several studies focused on functional characterization of the variable impact of genetic alterations in a syndromic condition [8,9,10]. The experiments in these studies utilizing the PM-M technology analyzed blood-derived cells from different subsets of a larger cohort of individuals with Phelan–McDermid Syndrome (PMS) compared to a control group of 50 individuals. PhenoMetaboDiff was used to analyze the metabolic data produced from the Biolog PM-M plates M1 to M8 and a custom tryptophan plate. Jain et al. (2022) [8] were able to correlate significant differences in the metabolic profile between individuals with PMS who experienced seizures versus individuals with PMS who did not experience seizures. Moffitt et al. (2023a) [9] were able to further the previous study on the same group of individuals with PMS who experienced sleep disturbances versus those who did not. PhenoMetaboDiff was able to analyze the data to show that individuals with PMS, in general, had a distinct significant decrease in the metabolism of energy sources on M1. The sleep disturbance group showed an abnormally increased metabolic response to ionic species found on M5, whereas the group not experiencing sleep disturbances showed decreased metabolic response to hormones found on M6. These results indicate that the differences in a PMS population and their metabolic profiles suggest pathogenic mechanisms affecting particular pathways in each group (sleep disturbances and no sleep disturbances) [9]. Lastly, PhenoMetaboDiff has been utilized to stratify a PMS population based on the metabolic response to specific compounds, such as Insulin-like growth factor-1 (IGF-1) and human growth hormone (hGH) [10]. This stratification of a population based on a response to a specific compound allows for an investigation into pathogenic mechanisms, identification of biomarkers, exploration of responses to candidate drugs in in vitro and in vivo models, and eventually, the selection of better candidates for clinical trials. Current in vivo studies are being conducted utilizing patient selection based on the PhenoMetaboDiff program.

## 4. Discussion

Metabolomic platforms, such as the OmniLog^®^ PM-M system, generate a vast amount of raw data that require complex analyses and remarkable expertise for the utilization of specific statistical approaches. Recently, Vehkala et al. [27] proposed a novel statistical pipeline to address the problem of the complexity of the analysis of PM data, and although their method has been developed on non-mammalian cells, it could be applied to the PM-M arrays. However, this pipeline does not address the need for a software interface to extrapolate the data from the OmniLog^®^ system and still requires high-level statistical expertise for its correct application. We introduced a package, PhenoMetaboDiff, which is a comprehensive open-source software for the identification, statistical analysis, and visualization of the Phenotype Mammalian MicroArray (OmniLog^®^ PM) system. A previous package in R, OPM, also offers tools for storing and analyzing the phenotype microarray data, but our package is a substantial improvement in functionality and usability. PhenoMetaboDiff is built on Shiny in R with a simple, user-friendly interface that allows users with limited computational capabilities to obtain results quickly. The package includes three distinct modules, each with its utility. The first module, PhenoDiff, uses the non-parametric Mann–Whitney U-test to identify significant differences between two groups of comparisons—in our case, patients and controls. The second module, Kinetic Analysis, plots a kinetic profile of substrate utilization over time. These kinetic values afford the construction of metabolic profiles of individual substrates over time. The user-defined features can visualize absorbance differences/overlaps between two groups by either selecting specific wells or by customizing the interval time. The final module, the Slope and AUC Calculator calculates descriptive curve parameters, lag phase, steepness of the slope, maximum curve height, and area under the curve (AUC) using the R package OPM. Above all, PhenoMetaboDiff provides a graphical user interface allowing the user to carry out their desired analysis using only simple points and clicks. Even though familiarity with RStudio might be helpful, users are not required to know R scripting to use our package. The user interface makes a quick and agile analysis of the microarray data possible, which will facilitate research and improve the efficiency of related research works. Moreover, due to the package being open-source and the simplicity of the implementation, along with the plethora of documentation available online for Shiny, it is easy for users with R coding experience to customize or extend the tool’s capabilities if the user was so inclined. As an instance, for future work, we will add more visualization modules based on Shiny that can be fully customized by users with only points and clicks to improve the compatibility of PhenoMetaboDiff for different microarray data. We will add support for other data formats, like YAML and JSON. Also, a more comprehensive and detailed comparison tool will be included in our package to further extend the functionality of PhenoMetaboDiff. Additionally, some more selections of statistical methods will be offered to users for different types of tests.

## 5. Conclusions

The growing field of omic technologies has amplified the opportunities for high-throughput investigations of both genetic and complex disorders. The advantages of generating a large amount of data are limited by the complexity of the analyses necessary for their interpretation and translation in research or clinical practice. Among the several omic-based technologies, the Biolog OmniLog^®^ PM-M system allows exploring the energy production of mammalian cells in different metabolic environments, generating a vast array of data encompassing hundreds of metabolites and pathways. The PhenoMetaboDiff software offers a user-friendly tool for the rapid and efficient analysis and interpretation of PM-M data, giving access to vast metabolomic data to a larger number of researchers and clinicians. A broader utilization of the OmniLog^®^ PM-M system can facilitate the investigation of the pathogenic mechanisms of both genetic and complex disorders, implement the development of metabolic biomarkers, and accelerate the generation of novel and more precise treatments.

## Data Availability

Data available in a publicly accessible repository: https://anonymous.4open.science/r/phenoMetaboDiff-62D4.
